# Dietary supplementation with probiotics promotes weight loss by reshaping the gut microbiome and energy metabolism in obese dogs

**DOI:** 10.1128/spectrum.02552-23

**Published:** 2024-01-25

**Authors:** Anna Kang, Min-Jin Kwak, Daniel Junpyo Lee, Jeong Jae Lee, Min Kyu Kim, Minho Song, Minjee Lee, Jungwoo Yang, Sangnam Oh, Younghoon Kim

**Affiliations:** 1 Department of Agricultural Biotechnology and Research Institute of Agriculture and Life Science, Seoul National University, Seoul, South Korea; 2 Institute of Agricultural Science and Technology, Kyungpook National University, Daegu, South Korea; 3 Division of Animal and Dairy Science, Chungnam National University, Daejeon, South Korea; 4 Ildong Bioscience, Pyeongtaek-si, Gyeonggi-do, South Korea; 5 Department of Functional Food and Biotechnology, Jeonju University, Jeonju, South Korea; School of Life Sciences, Nanchang University, Nanchang, China

**Keywords:** canines, pet probiotics, anti-obesity, *Enterococcus*, *Bifidobacterium*, pyruvate metabolism, glycolysis

## Abstract

Obesity and overweight among companion animals are significant concerns, paralleling the issues observed in human populations. Recent research has highlighted the potential benefits of various probiotics in addressing weight-related changes, obesity, and associated pathologies. In this study, we delved into the beneficial probiotic mechanisms in high-fat-induced obese canines, revealing that *Enterococcus faecium* IDCC 2102 (IDCC 2102) and *Bifidobacterium lactis* IDCC 4301 (IDCC 4301) have the capacity to mitigate the increase in body weight and lipid accumulation in obese canines subjected to a high-fat diet and hyperlipidemic *Caenorhabditis elegans* (*C. elegans*) strain VS29. Both IDCC 2102 and IDCC 4301 demonstrated the ability to reduce systemic inflammation and hormonal disruptions induced by obesity. Notably, these probiotics induced modifications in the microbiota by promoting lactic acid bacteria, including *Lactobacillaceae*, *Ruminococcaceae*, and *S24-7*, with concomitant activation of pyruvate metabolism. IDCC 4301, through the generation of bacterial short-chain fatty acids and carboxylic acids, facilitated glycolysis and contributed to ATP synthesis. Meanwhile, IDCC 2102 produced bacterial metabolites such as acetic acid and butyric acid, exhibiting a particular ability to stimulate dopamine synthesis in a canine model. This stimulation led to the restoration of eating behavior and improvements in glucose and insulin tolerance. In summary, we propose novel probiotics for the treatment of obese animals based on the modifications induced by IDCC 2102 and IDCC 4301. These probiotics enhanced systemic energy utilization in response to high caloric intake, thereby preventing lipid accumulation and restoring stability to the fecal microbiota. Consequently, this intervention resulted in a reduction in systemic inflammation caused by the high-fat diet.

**IMPORTANCE:**

Probiotic supplementation affected commensal bacterial proliferation, and administering probiotics increased glycolysis and activated pyruvate metabolism in the body, which is related to propanate metabolism as a result of pyruvate metabolism activation boosting bacterial fatty acid production via dopamine and carboxylic acid specialized pathways, hence contributing to increased ATP synthesis and energy metabolism activity.

## INTRODUCTION

Due to demographic shifts, including increased economic prosperity and particularly during the ongoing epidemic, the global prevalence of companion animals has experienced rapid growth. Presently, it is estimated that roughly one-third of households worldwide own a pet. With the escalating number of individuals sharing their living spaces with pets, the relationship between humans and their animal companions has evolved beyond mere ownership. As implied by the term “companion” animals, contemporary society perceives pets as more than possessions—they are regarded as friends or family members. In response to this evolving perspective, the pet industry has adapted, reflecting the heightened desire of individuals to invest more quality time with their cherished animal companions (1, 2).

In contrast to wild animals contending with threats from other species and unpredictable environmental conditions, the mortality of domesticated animals is predominantly attributed to physical factors. Among young animals, mortality is most commonly attributed to trauma and infectious diseases, while elderly animals often succumb to chronic ailments like cancer, diabetes, and obesity. Much like the recognition of metabolic disorders as a primary factor in various chronic human diseases, pet obesity has been identified as a contributor to health disparities. Recent studies focusing on canines reveal alarming statistics, with 29%–34% of dogs classified as overweight and 5%–8% diagnosed as obese. These findings underscore the prevalence of pet obesity and its potential impact on the health and well-being of companion animals, mirroring the concerns associated with metabolic disorders in human health (3–5).

While the complete etiology of obesity remains not fully elucidated, its fundamental cause lies in the accumulation of lipids. Domesticated animals typically become obese due to factors such as restricted physical activity and excessive energy intake, often stemming from the consumption of high-calorie foods, particularly indulgent treats. In the United States, where pet ownership is prevalent, food ranks among the most substantial expenses for pet owners. Both feline and canine owners invest significantly in standard pet food, with additional expenditures on treats. Concurrently, expenses on toys, accessories, and other pet-related items contribute to the continuous rise in overall pet-related costs. Projections indicate a gradual increase in spending on pet food and treats in the United States. In response to the rising awareness of pet obesity and the associated dietary restrictions imposed by owners, manufacturers of pet probiotics have discerned the evolving needs of pet owners. This recognition has prompted a focus on developing products that align with the dietary requirements of obese pets, reflecting a growing market demand for specialized pet nutrition (6–8).

The choice of *Caenorhabditis elegans* as the model organism in this study was motivated by its numerous practical advantages for screening potential probiotics. In comparison to other animal models, *C. elegans* boasts a relatively short lifespan, lasting approximately 3–4 weeks, and is easy to maintain, undergoing quick reproductive cycles within a matter of days. Notably, *C. elegans* shares 65% of genes associated with human diseases and possesses intestinal cells that exhibit structural similarities to those found in the human intestine (9, 10). The transparency of *C. elegans*’ body provides a distinct advantage, allowing for easy observation of lipid droplets and other potentially hazardous accumulations. This characteristic makes *C. elegans* a preferred model for investigating various aspects of health, including obesity, feeding behaviors, satiety, and metabolic disorders (11, 12). The combination of these attributes positions *C. elegans* as a practical and informative model for the initial screening of potential probiotics.

A complex microbial community resides in the gastrointestinal tract of animals, and healthy canines and felines have been demonstrated to exhibit an abundance of *Bifidobacterium*, *Lactobacillus*, *Enterococcus*, *Mogibactera*, *Clostridium*, and more (13–15); clinical trials of those canine- and feline-origin probiotics were successful in improving the digestibility and overall health of senior dogs (16, 17), relieving diarrhea (18), lowering serum glucose and cholesterol levels, and decreasing systemic inflammation (19, 20).

Given the substantial impact of gut microbiota on host systemic health, recent investigations have unveiled a correlation between gut microbiota composition and obesity. Typically, obese individuals exhibit an elevated presence of gram-positive bacteria, such as *Firmicutes*, coupled with a reduction in gram-negative bacteria, particularly *Bacteroidetes* (21, 22). Additionally, it has been observed that obese individuals tend to possess a less diverse gut microbiota compared to their non-obese counterparts. Supplementation of obese individuals with *Lactobacillus*-fermented dairy products has demonstrated a reduction in serum cholesterol and systemic cytokines associated with diabetes (23–25).

This reduction in harmful factors suggests a modulation of gut microbiota through probiotic administration. Antibiotics have also been explored as an adjunct in obesity treatment, highlighting the intricate interplay between gut microbiota and metabolic health. Probiotics exert their beneficial effects by generating microbial byproducts, such as vitamin B and short-chain fatty acids, through the fermentation of indigestible carbohydrates in the gastrointestinal tract. These byproducts serve as an energy source for adenosine triphosphate (ATP) production via the citric acid cycle (26, 27).

While the positive impact of probiotics on host health, including their role in obesity prevention, is acknowledged, a comprehensive understanding of their mechanisms is still evolving. Studies on probiotic effects in obese individuals remain primarily confined to human research, and the precise mechanisms underlying probiotic-induced modulation of host health in both humans and animals are not fully elucidated. Consequently, this study seeks to explore how probiotics, specifically *Enterococcus faecium* IDCC 2102 and *Bifidobacterium lactis* IDCC 4301, regulate gut microbiota to mitigate obesity in obese beagles and the hyperlipidemic *C. elegans* strain VS29.

## RESULTS

### Probiotic treatment upregulated the lifespan of *C. elegans* and prevented lipid accumulation

**Fig 1:**
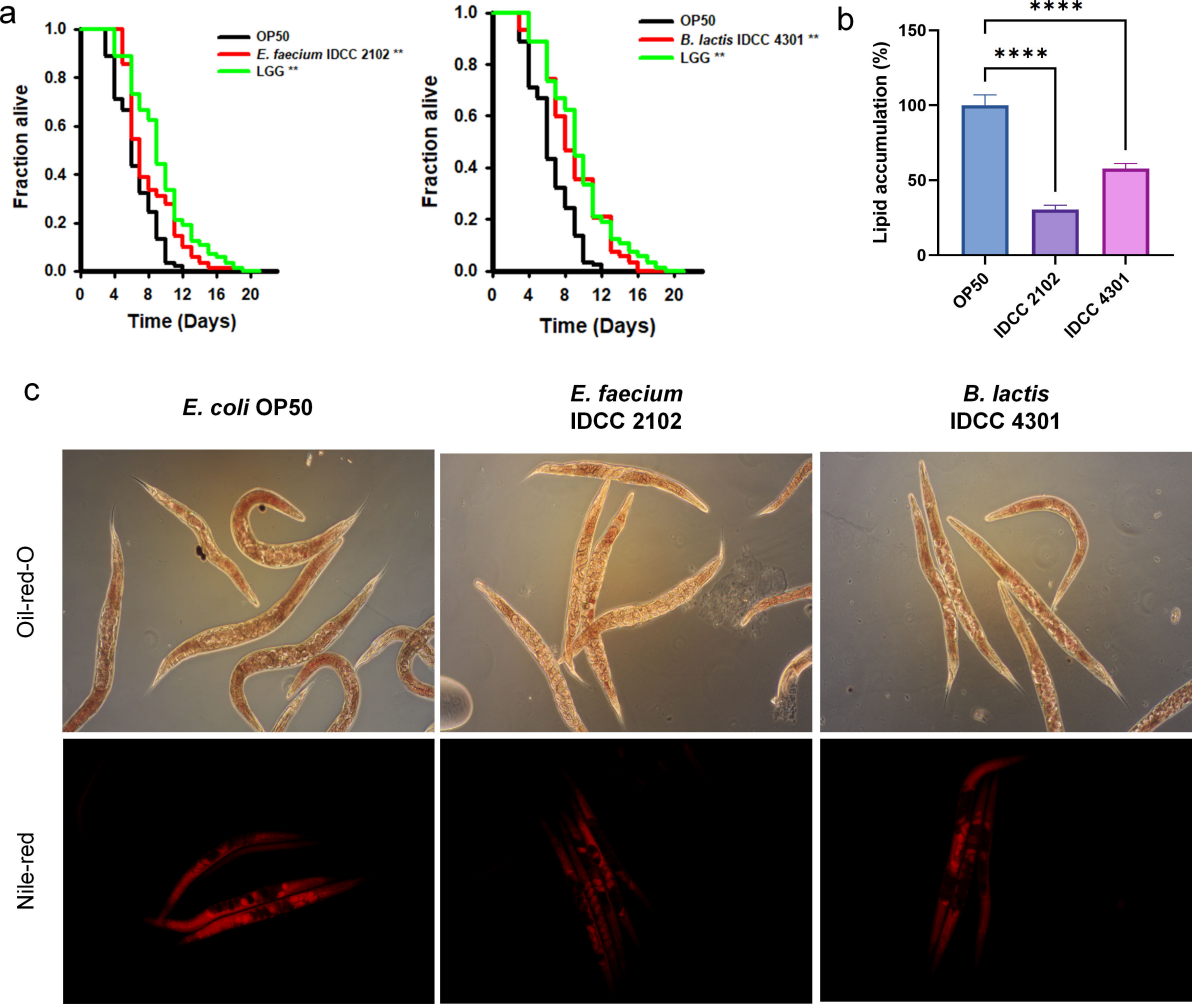
Probiotic treatment of *C. elegans* enhanced longevity and prevented lipid accumulation. The effects of *E. faecium* IDCC 2102 and *B. lactis* IDCC 4301 on the lifespan of *C. elegans fer-15;fem-1* were evaluated (**a**). The lipid content of *C. elegans* VS-29 was evaluated through oil red O staining (upper) and Nile red staining (lower) after 48 h of exposure to *E. coli* OP50, IDCC 2102, and IDCC 4301 (*n* = 10 per group, each experiment was performed three times) (**c**). The fluorescence of each trial was analyzed using i-Solution software. Data are shown as the means ± SEMs; significant differences were determined using one-way ANOVA at ^**^P < 0.01, ^***^P < 0.001, and ^****^P < 0.0001 compared with *E. coli* OP50.

*C. elegans* lifespan analysis was utilized to evaluate the probiotic capability of *E. faecium* IDCC 2102 and *B. lactis* IDCC 4301. *C. elegans* is a well-established genetic model with the advantage of relatively simple genetic editing and a shorter life expectancy than other animal models (28). Both *E. faecium* IDCC 2102 and *B. lactis* IDCC 4301 extended the longevity of *C. elegans* in comparison to *Escherichia coli* OP50 (OP50), and IDCC 2102 elevated the longevity even more than *Lacticaseibacillus rhamnosus* GG (LGG), one of the most clinically renowned probiotics worldwide (29) (Fig. 1a). Next, the lipogenic inhibitory capacity of *E. faecium* IDCC 2102 and *B. lactis* IDCC 4301 was evaluated by lipid staining with the hyperlipidemic *C. elegans* VS29 strain. The lipid content was determined by oil red O and Nile red staining; *E. faecium* IDCC 2102 and *B. lactis* IDCC 4301 treatment significantly decreased the lipid accumulation of VS29 stains (Fig. 1b and c).

Furthermore, to assess the efficacy of the probiotic intervention in reducing lipid accumulation in the hyperlipidemic *C. elegans* model, a metabolite analysis was conducted. Significantly, the hyperlipidemic control group exhibited elevated levels of L-serine, L-alanine, and hydroxyproline, key components of glycine. Normally, glycine plays a crucial role in the regulation of food consumption and glucose homeostasis by promoting insulin and GSH (glutathione) production. However, excess amino acids are known to be converted into fat and stored in fat depots; alternatively, they can be utilized for glucose synthesis through gluconeogenesis. In the context of *C. elegans*, an excess of glycine proves detrimental to its longevity, as the organism heavily relies on a tightly regulated insulin/IGF-1 signaling pathway (30–32). Consequently, it appears that *E. faecium* IDCC 2102 and *B. lactis* IDCC 4301 may enhance the longevity of *C. elegans* by modulating glycine synthesis (Fig. 1a; Fig. S1b and c).

The hyperlipidemic *C. elegans* strain VS29 (cont) displayed elevated levels of factors associated with fatty acid metabolism, such as L-gluconolactone and oxalic acid, alongside reduced levels of L-tryptophan. Additionally, the accumulation of glycine in tissues, particularly the brain, can disrupt normal brain functions. Glycine serves as an inhibitory neurotransmitter capable of suppressing dopamine (31, 33, 34). This pattern was reversed following treatment with *E. faecium* IDCC 2102 and *B. lactis* IDCC 4301, as they increased the levels of beneficial bacterial short-chain acids such as 5-aminovaleric acid and acetic acid, as well as the serotonin precursor L-tryptophan. Simultaneously, they decreased the levels of glycine and its precursor molecules. This outcome suggests that probiotic treatment indeed proves effective in the hyperlipidemic *C. elegans* model, exerting a regulatory effect on lipid accumulation and neurotoxicity (Fig. S1).

### Probiotic treatment alleviated the obesogenic features of canines fed a HFD

**Fig 2:**
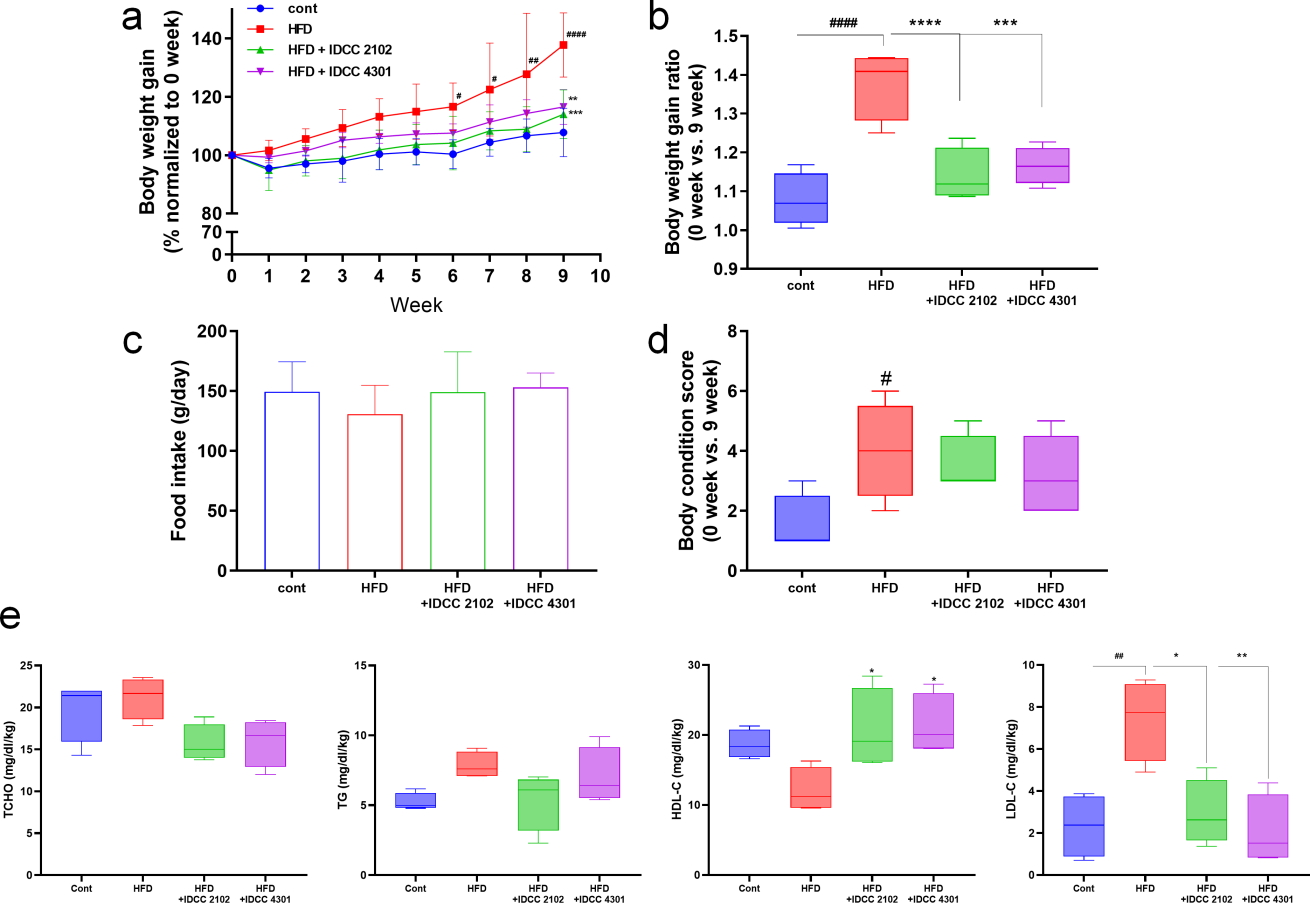
Probiotic treatment alleviated body weight gain and lipid accumulation in HFD-induced obese canines. To investigate the physiological changes induced by treatment with *Enterococcus faecium* IDCC 2102 (IDCC 2102) and *Bifidobacterium lactis* IDCC 4301 (IDCC 4301) in obese canines, body weight gain (**a and b**) and food intake were measured weekly (**c**). After 9 weeks of HFD and probiotic treatment, individual body condition scores were analyzed (**d**). The lipid metabolism markers were analyzed from serum; total cholesterol (TCHO), triglyceride (TG), high-density lipoprotein-C (HDL-C), and low-density lipoprotein-C (LDL-C) were adjusted by individual body weight (**e**). Cont, normal chow group; HFD, high-fat diet group. Data are shown as the means ± SEMs (*n* = 3 per group); significant differences were determined using one-way ANOVA at ^#^*P* < 0.05, ^##^*P* < 0.01, and ^####^*P* < 0.0001 compared with cont; ^**^P < 0.01, ^***^P < 0.001, and ^****^P < 0.0001 compared with HFD.

Canines were fed a high-fat diet to induce obesity for 9 weeks, during which *E. faecium* IDCC 2102 and *B. lactis* IDCC 4301 were administered concurrently (10^10^ CFU/day) to determine the anti-obesity impact of these two probiotics. As anticipated, the body weight of the high-fat diet (HFD) group without probiotics increased and, beginning in week 6, was considerably higher than that of the control group (cont) fed normal chow. At the end of the experiment, the HFD group weighed 127.8% ± 10.2% more than the control group. However, probiotic treatments altered the features. Even with a high-fat diet, probiotic treatments significantly reduced the body weight gain ratio. Compared to the HFD group, *E. faecium* IDCC 2102 and *B. lactis* IDCC 4301 significantly decreased the final body weight by 17.3% ± 6.0% and 15.4% ± 4.3%, respectively (Fig. 2a and b). In addition, the body condition score based on body shape exhibited significant lipid accumulation in the HFD group and improvement in the probiotic-treated groups (Fig. 2d; Fig. S2). Given that the amount of food consumed did not differ significantly regardless of probiotic therapy (Fig. 2c), probiotic treatment could prevent lipid accumulation.

The serum was then subjected to DRI-CHEM analysis to determine the various lipid levels standardized by individual body weight, as individual body weight varied. TCHO was increased by 4.7% ± 12.3% in the HFD group compared to the control, and *E. faecium* IDCC 2102 and *B. lactis* IDCC 4301 decreased the TCHO level by 26.1% ± 10.5% and 24.9% ± 13.6% compared to the HFD group. TG, a store lipid, was increased by 50.2% ± 17.9% in the HFD group compared to the control group, whereas *E. faecium* IDCC 2102 and *B. lactis* IDCC 4301 treatment reduced TG levels by 31.5% ± 26.9% and 10.4% ± 25.7%, respectively. HDL-C, which inhibits cholesterol biosynthesis, was decreased by 35.4% ± 17.0% in the HFD group and increased by 71.7% ± 47.6% and 77.4% ± 356.2% in the *E. faecium* IDCC 2102 and *B. lactis* IDCC 4301 groups, respectively. In the same context, the ratio of LDL-C, which transfers cholesterol to the periphery, increased by 218.9% ± 82.6% in the HFD group compared to the control group, while it decreased by 60.5% ± 21.1% and 72.3% ± 22.6% in the *E. faecium* IDCC 2102 and *B. lactis* IDCC 4301 groups.

### Probiotic treatment modulated inflammatory cytokines

**Fig 3:**
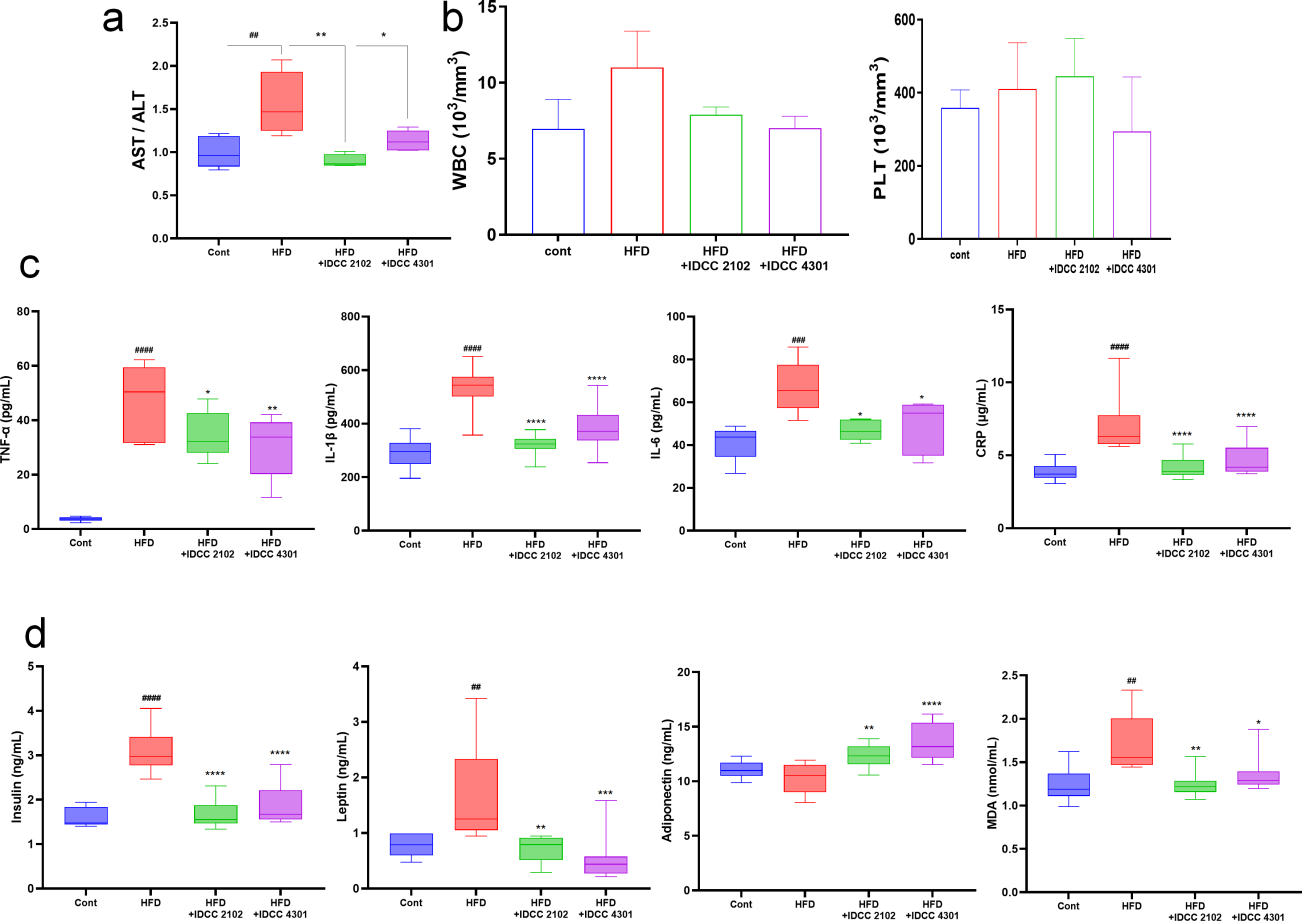
Probiotic treatment reduced inflammatory markers and restored lipid metabolism in blood and serum. Serum AST concentration was adjusted with the ALT level, (**a**) and white blood cell (WBC) and platelet (PLT) levels were analyzed from whole blood samples (**b**). Moreover, inflammatory cytokines (**c**) and energy metabolism regulators (**d**) were examined in serum by an enzyme-linked immunosorbent assay (ELISA). Data are shown as the means ± SEMs (*n* = 3 per group); significant differences were determined using one-way ANOVA at ^##^*P* < 0.01, ^###^*P <* 0.001, and ^####^*P* < 0.0001 compared with cont; ^*^P < 0.05, ^**^P < 0.01, ^***^P < 0.001, and ^****^P < 0.0001 compared with HFD.

Lipid accumulation could induce sensitization of the immune system, which could lead to chronic inflammation in the long term. Therefore, inflammatory markers were analyzed in serum and whole blood samples. To investigate the systemic inflammatory marker, aspartate aminotransferase (AST) was normalized by alanine aminotransferase (ALT); the AST/ALT level was significantly increased in the HFD group by 40.9% ± 10.3% compared to the normal chow control group, and *E. faecium* IDCC 2102 and *B. lactis* IDCC 4301 treatment reduced the AST/ALT level by 40.8% ± 6.0% and 27.8% ± 11.6%, respectively (Fig. 3a).

The term CBC stands for complete blood counting, and it is a means of evaluating general health for various illness conditions, such as infection, by validating the amount of active cells in the blood. WBC count and PLT count elevations represent immune disorders and an acute inflammatory state. During the experiment, CBC tests were taken with blood for 9 weeks. The results of these measurements showed that all of the test subjects were within the normal range. Nevertheless, when compared to the control group, the WBC level increased by 39.9% ± 37.3% in the HFD group and reduced by 22.9% ± 10.8% in the IDCC 2102 and 23.3% ± 10.1% in the IDCC 4301 treatment group. Specifically, the IDCC 4301 treatment group confirmed that the PLT count inflammatory factor was reduced by 28.3% ± 36.3% in comparison to the HFD group (Fig. 3b).

To ensure that the probiotics employed in this trial had an anti-inflammatory effect, serum inflammatory markers were identified using an ELISA. Treatment with *E. faecium* IDCC 2102 and *B. lactis* IDCC 4301 changed the immune response by lowering the levels of the pro-inflammatory adipokines TNF-α and IL-1β by a large amount. They also lowered the level of IL-6 by a large amount, which helped restore the hormonal balance needed for adipocyte differentiation and lipid metabolism (Fig. 3c). Furthermore, the administration of probiotics significantly improved insulin sensitivity as well as the levels of leptin and adiponectin, which are involved in the suppression of fat storage by balancing the intake/consumption of energy in the body as well as hunger regulation (35) (Fig. 3d).

### Probiotic treatment altered the microbial composition of obese canines

**Fig 4:**
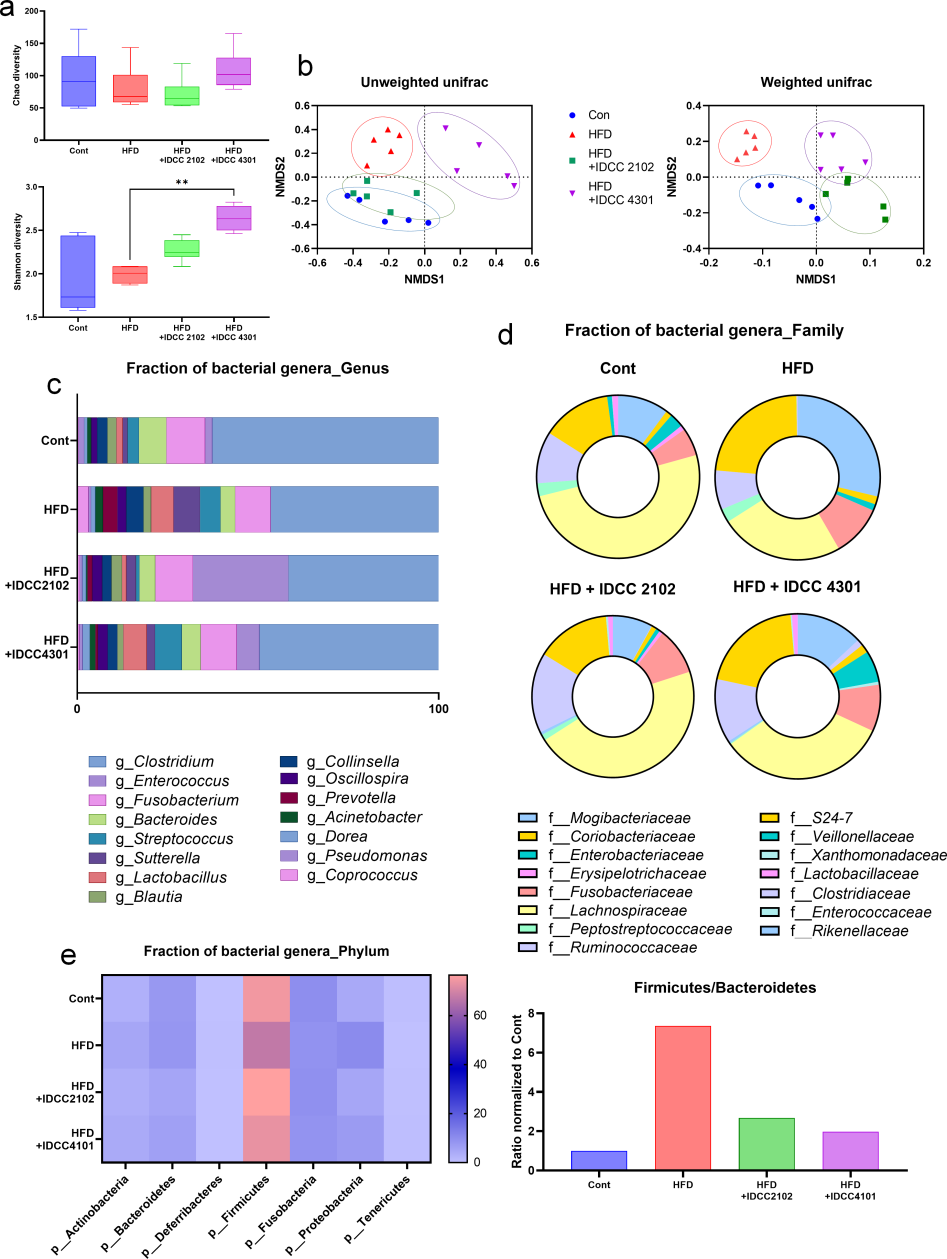
The microbiota of HFD-induced obese canines was diversified by probiotic treatment. Metagenomic studies enable researchers to investigate the relative abundance of the microbiome. The Chao and Shannon index values represent the alpha diversity of the microbiome from the fecal samples of each group (**a**). Data are shown as the means ± SEMs (*n* = 3 per group); significant differences were determined using one-way ANOVA at ^**^P < 0.01. PCoA plots are spotted based on weighted and unweighted UniFrac distances of the fecal microbiome of each group with distinct clusterings (**b**). The metagenomics analysis indicated different compositions of the top 15 abundant bacterial genera of the genus (**c**), family (**d**), and phylum (**e**) of each group.

A metagenomic analysis of canine feces from each group was performed to examine the microbial shift after probiotic administration. NGS (next generation sequencing) analysis was used to sequence the V4 region of 16S rRNA. A total of 9,604 OTUs were examined, and 301 of them were identified as bacteria with >97% identification reliability using the NCBI database. To assess the diversity among groups, alpha diversity was analyzed; the Chao index and Shannon index measure the number of species and effective number of species, respectively. Although there was no significant difference between groups in the Chao index, *B. lactis* IDCC 4301 demonstrated high variety when compared to the HFD group in the Shannon index (Fig. 4a). In addition, weighted and unweighted beta-diversity analyses revealed that each probiotic treatment resulted in unique microbiota composition clustering (Fig. 4b).

As observed earlier, it is evident that diet can influence microbial composition. In contrast to the regular chow control group, the HFD-induced obese beagles experienced a reduction of 3.9% in their genus diversity, namely, *Enterococcus*, *Bacteroides*, and *Lactobacillus*. On the other hand, *E. faecium* IDCC 2102 treatment restored *Enterococcus*, *Fusobacterium*, and *Bacteroides* genus levels, and *B. lactis* IDCC 4301 treatment increased *Fusobacterium*, *Bacteroides*, and *Lactobacillus* at the genus level (Fig. 4c). The microbial changes at the genus level were reflected at the family level. *E. faecium* IDCC 2102 treatment particularly increased the composition of *Lachnospiraceae*, *Enterobacteriaceae*, and *Lactobacillaceae,* and *B. lactis* IDCC 4301 treatment specifically increased *Ruminococcaceae*, *S24-7*, and *Lactobacillaceae* at the family level compared to the HFD-induced obese canines (Fig. 4d). Moreover, the Firmicutes/Bacteroides ratio was exceptionally high in the HFD group, which was not surprising given that prior research has suggested this ratio as a potential biomarker of obesity (Fig. 4e).

### Metabolomic profile changes made by probiotic treatment

**Fig 5:**
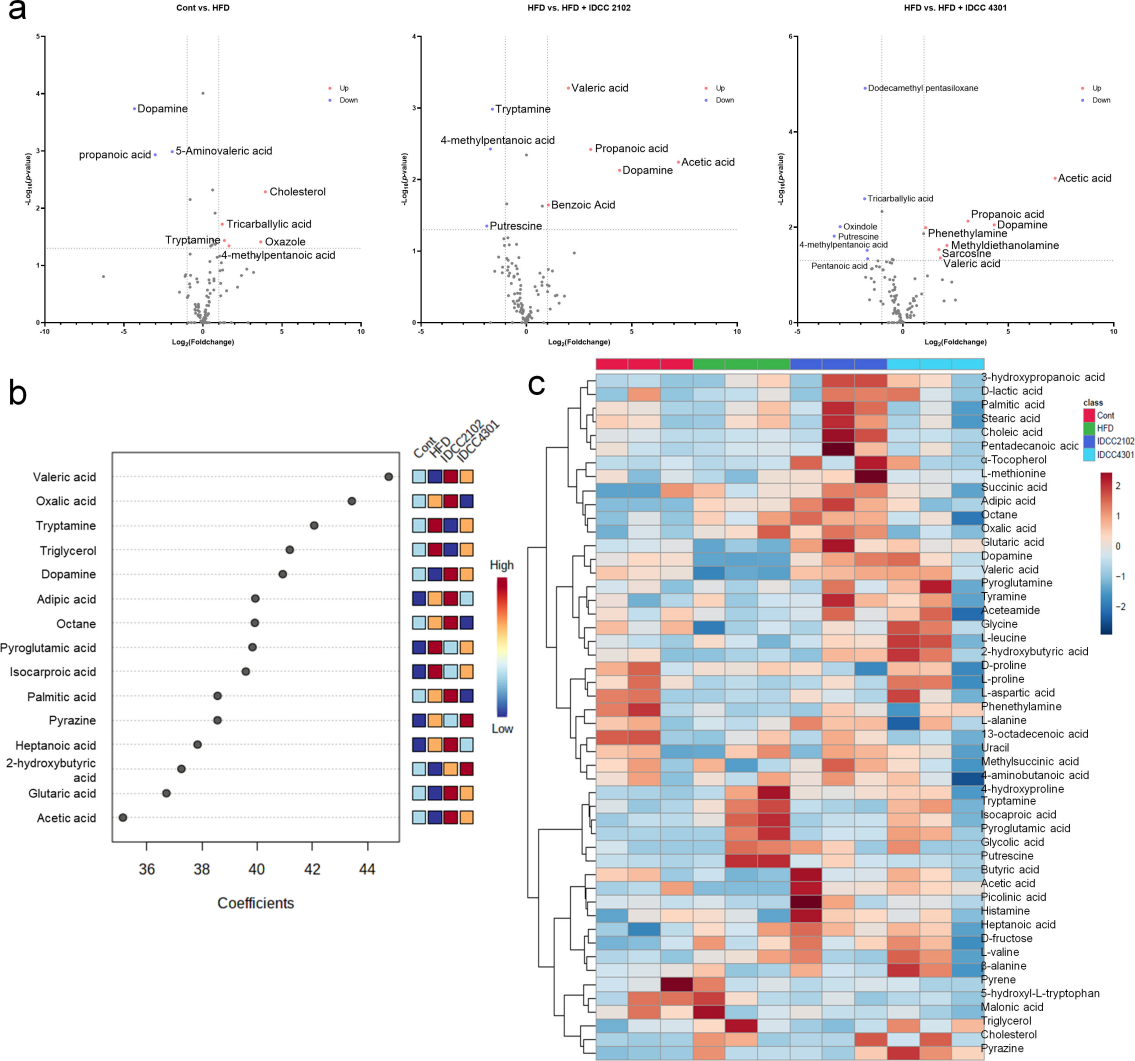
The microbiota influenced by probiotics produced unique metabolites. After 9 weeks of feeding, fecal samples of obese canines were collected for metabolomic analysis. The dots in volcano plot graphs indicate the bacterial metabolites that were significantly increased (red; fold change > 1, *P* < 0.05) or decreased (blue; fold change < 1, *P* < 0.05) (**a**). The weighted sum of absolute regression coefficients is represented by partial least squares, and the colored boxes indicate the concentrations of the relevant metabolite in each group (**b**). The heatmap exhibits the top 50 abundant metabolites of each group; different colors represent the concentration (**c**).

As the composition of the microbiome varied based on the diet of each group, it was discovered that the bacterial metabolites were also quite distinct. Obese canines produced higher levels of histamine, malonic acid, picolinic acid, methylsuccinic acid, and 5-hydroxy-L-tryptophan levels than the normal chow control group, although tyramine synthesis was decreased. Interestingly, treatment with *E. faecium* IDCC 2102 and *B. lactis* IDCC 4301 resulted in the majority of the elevated metabolites in the HFD group being downregulated. Tyramine, alpha-tocopherol, tryptamine, D-proline, and stearic acid were all upregulated in the *E. faecium* IDCC 2012 group, while methylsuccinic acid, picolinic acid, 5-hydroxy-L-tryptophan, acetic acid, butyric acid, L-valine, and histamine were all downregulated. *B. lactis IDCC* 4301 treatment specifically decreased malonic acid and phenethylamine while elevating isocaproic acid, adipic acid, succinic acid, and beta-alanine (Fig. 5a).

### Metabolic pathway modulated by *E. faecium* IDCC 2102

**Fig 6:**
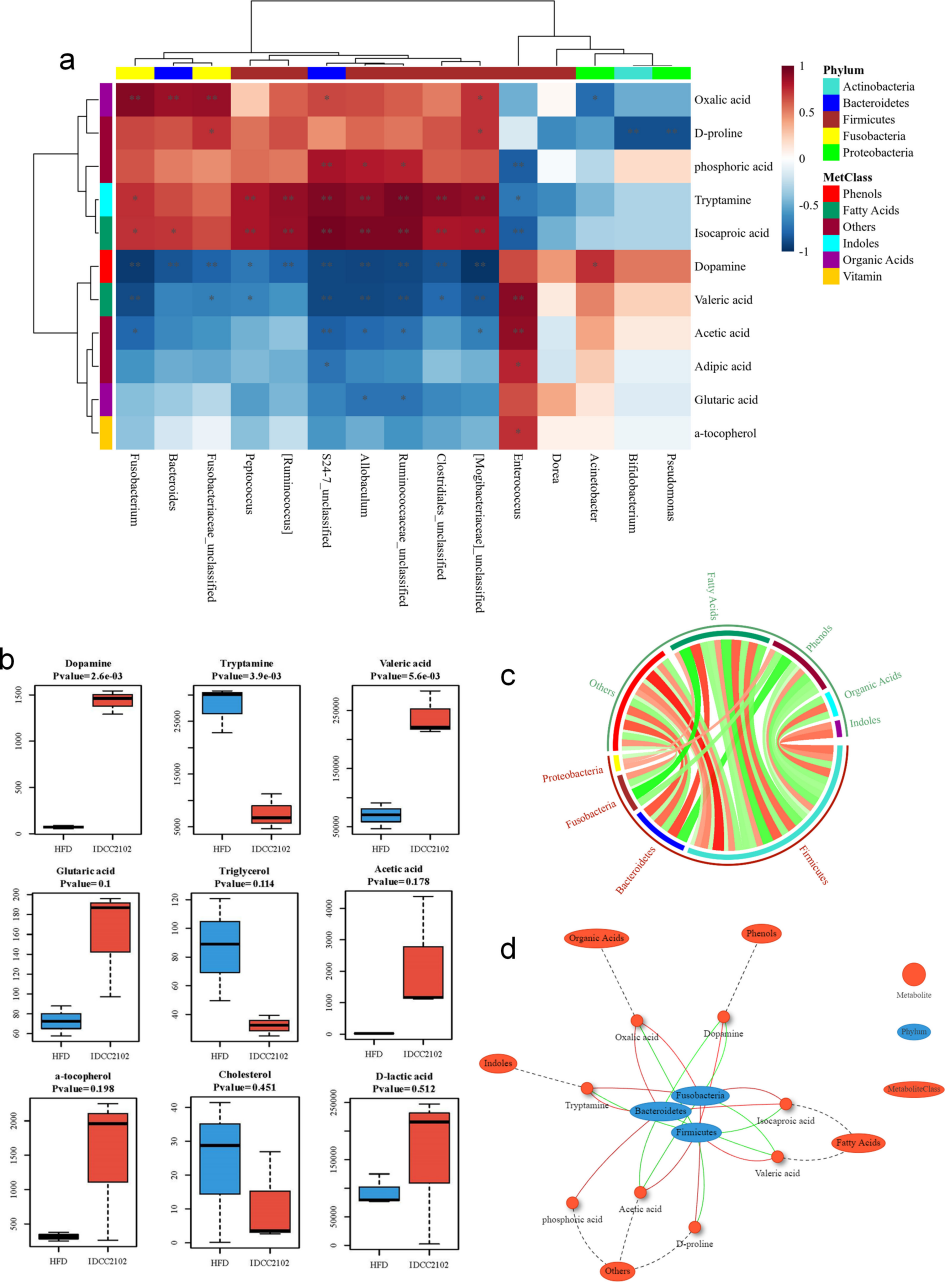
The correlation between the microbiota and metabolites produced by the administration of *E. faecium* IDCC 2102. The heatmap depicts the SparCC correlation efficiency between the microbiota composition and IDCC 2102 group metabolites (**a**). The darker color represents the higher concentrations of metabolites. The boxplots depict the most dissimilar metabolites between the HFD and IDCC 2102 groups; mean (SD) values of each group are provided by Student’s *t*-test, and the median (Interquartile range; IQR) values are provided by the Mann‒Whitney *U* test (**b**). Circos plots exhibit SparCC correlations between microbiota and metabolites; red and green lines represent positive and negative correlations (**c**) and the network of SparCC correlations (**d**).

Since bacterial metabolites are thought to be strongly related to the microbiome structure, we examined the interaction between microbiota and metabolites generated by each probiotic treatment by normalizing the metabolite levels by microbiome composition. Treatment with *E. faecium* IDCC 2102 stimulated the colonization of lactic acid bacteria, such as *Ruminococcus*, *Lactobacillus*, and *S24-7*, which led to a general increase in amino acids and short-chain fatty acids, such as oxalic acid, phosphoric acid, and isocaproic acid. In particular, the *Enterococcus* genus colonized and significantly elevated dopamine, valeric acid, acetic acid, and glutaric acid levels (Fig. 5b and c, Fig. 6a and b). Treatment with *E. faecium* IDCC 2102 increased the proportion of *Firmicutes*, *Bacteroides*, *Fusobacteria*, and *Proteobacteria*, resulting in the proportional synthesis of fatty acids, phenols, organic acids, and indoles (Fig. 6c and d).

### Metabolic pathway modulated by *B. lactis* IDCC 4301

**Fig 7:**
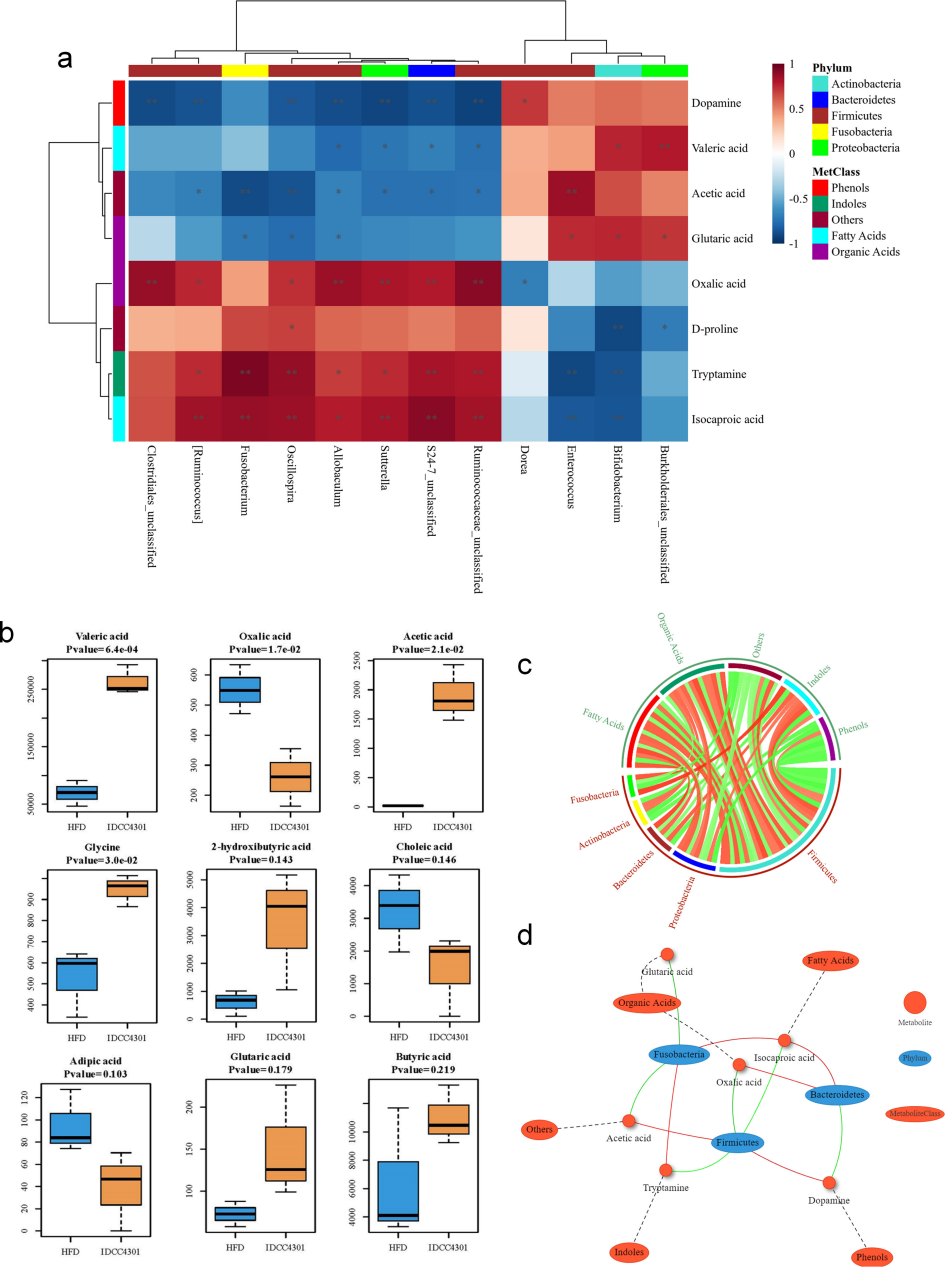
The correlation between the microbiota and metabolites produced by the administration of *B. lactis* IDCC 4301. The heatmap depicts the SparCC correlation efficiency between the microbiota composition and IDCC 4301 group metabolites (**a**). The darker color represents the higher concentrations of metabolites. The boxplots depict the most dissimilar metabolites between the HFD and IDCC 4301 groups; the mean (SD) values of each group are provided by Student’s *t*-test, and the median (Interquartile range; IQR) values are provided by the Mann‒Whitney *U* test (**b**). Circos plots exhibit SparCC correlations between microbiota and metabolites; red and green lines represent positive and negative correlations (**c**) and the network of SparCC correlations (**d**).

*B. lactis* IDCC 4301 exhibited somewhat different metabolomics results. *B. lactis* IDCC 4301 treatment also increased the colonization of general lactic acid bacteria and increased the general amino acids, but the colonization of *Bifidobacterium* increased the short- and medium-chain fatty acids such as isocaproic acid and amino acids such as adipic acid, succinic acid, and beta-alanine more dramatically (Fig. 5c and d, Fig. 7a and b). Treatment with *B. lactis* IDCC4301 increased the proportion of *Firmicutes*, *Proteobacteria*, *Bacteroidetes*, *Actinobacteria*, and *Fusobacteria*, resulting in the proportional synthesis of fatty acids, organic acids, indoles, and phenols (Fig. 7c and d).

### Functional pathways modulated by probiotic treatments

**Fig 8:**
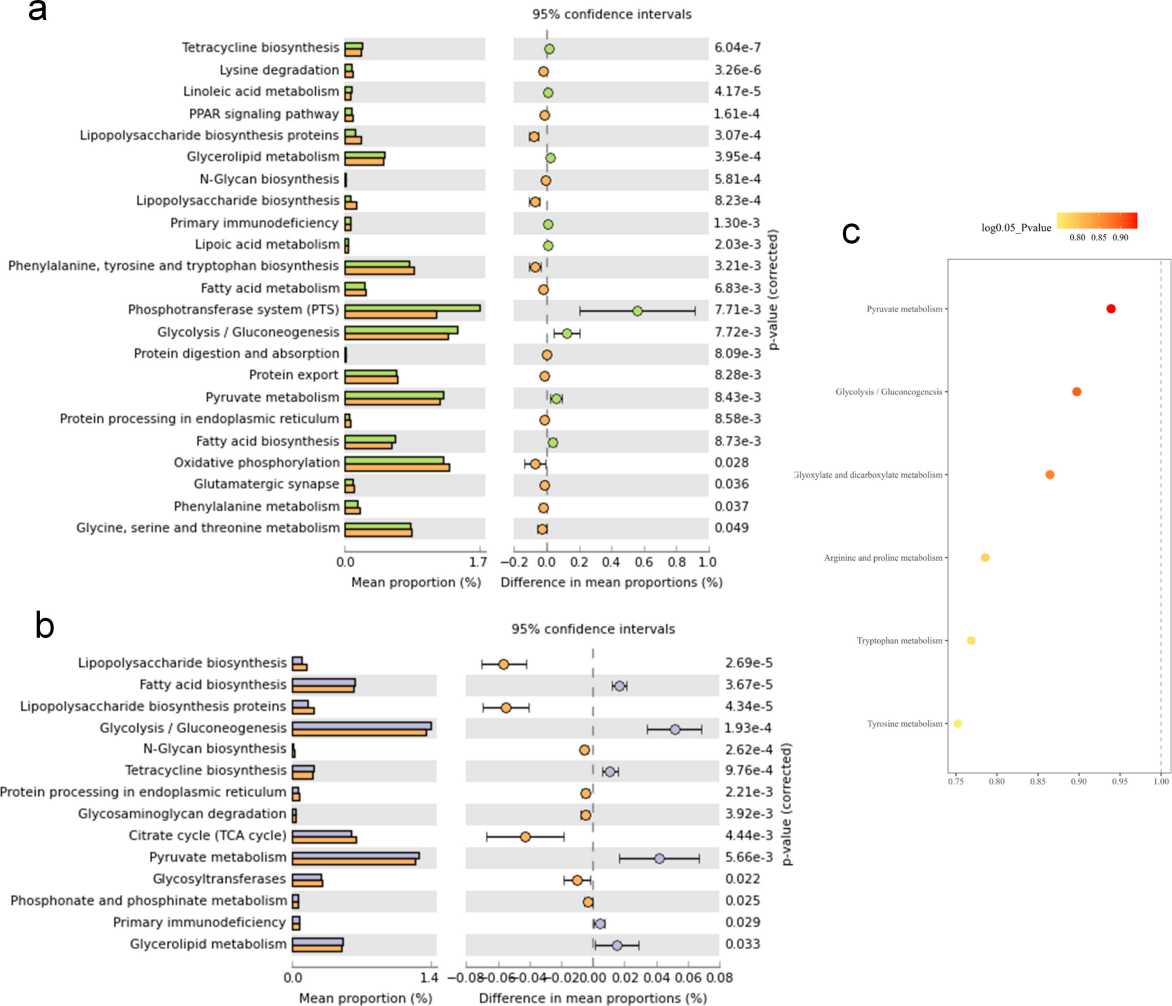
Comparative investigation of metabolic pathways between the HFD and probiotic-treated groups. Differential functional metabolic pathways of groups treated with HFD and probiotics are illustrated (**a and b**). Plots represent the significant metabolic pathways with a *P-value* of 0.05. KEGG pathway analysis provides a functional prediction of distinct metabolic pathways with each probiotic using a linear discriminant analysis approach. The intensities of color boxes denote significance directly, and *P-*values <0.05 were considered statistically significant (**C**).

Obesity induction and probiotic treatments modified the metabolic pathways. The HFD obese canine group significantly increased the functional and Kyoto Encyclopedia of Genes and Genomes (KEGG) pathways of aminoacyl-tRNA, fatty acid biosynthesis (valine, leucine, isoleucine, pantothenate, and CoA), and amino acid metabolism (arginine, proline, and tryptophan) (Fig. 8a and c). Probiotic treatments with *E. faecium* IDCC 2102 and *B. lactis* IDCC 4301 enhanced pyruvate metabolism, glucose metabolism (glycolysis and gluconeogenesis), energy production (glyoxylate and dicarboxylate metabolism), and amino acid metabolism (arginine and proline) (Fig. 8b and c).

## DISCUSSION

The gut microbiota, residing in the gastrointestinal tract, plays a crucial role in influencing the overall health of the host. Probiotics, which are live beneficial bacteria, have been utilized to preserve homeostasis, contributing to the rapid growth of the pet probiotics industry. However, there has been a relatively limited number of metagenomic and metabolomic studies conducted in animals compared to those in humans (36, 37). Consequently, the objective of this investigation was to ascertain how the administration of probiotics might regulate the obesity-induced disruption of the microbiota, stemming from an imbalance in energy intake and expenditure.

Initially, the probiotics *Enterococcus faecium* IDCC 2102 (IDCC 2102) and *Bifidobacterium lactis* IDCC 4301 (IDCC 4301) demonstrated a significant increase in the lifespan of *Caenorhabditis elegans* (*C. elegans*) when compared to the control *Escherichia coli* OP50 (OP50). Additionally, they effectively prevented lipid accumulation in *C. elegans*. This observed trend was further corroborated in canine models, where both IDCC 2102 and IDCC 4301 significantly curtailed weight gain in high-fat diet-treated canine subjects. Moreover, these probiotics restored lipase activity by reducing lipid-storing lipoproteins in the canine models, highlighting their potential in mitigating the effects of a high-fat diet.

Lipid accumulation due to obesity has been proven to burden blood flow and contribute to cardiovascular diseases, systemic inflammation, and hormonal disruption. As obesity develops, adipose tissue composed of adipocytes differentiates and transforms into highly active endocrine organs. They secrete pro-inflammatory adipokines and hunger hormones (35, 38) and induce insulin resistance (39). Both IDCC 2102 and IDCC 4301 significantly retained inflammatory cytokines and restored hormonal homeostasis compared to the high-fat diet-induced obese canine group (HFD).

Subsequently, we investigated the microbial changes induced by the treatment with IDCC 2102 and IDCC 4301. α-Diversity, which assesses microbial diversity within groups, and β-diversity, measuring microbial composition distances between groups (40), were employed for analysis. Notably, treatments with IDCC 2102 and IDCC 4301 led to significant alterations in the microbial community structure. An advantageous aspect of probiotics is their capacity to modulate gut microbial ecosystems by inhibiting pathogens and fostering the growth of beneficial commensal bacteria (41). The induction of obesity substantially increased the *Firmicutes*/*Bacteroidetes* ratio, a well-recognized characteristic of obesity. *Firmicutes* are known for their efficiency in extracting energy from food, suggesting their role in facilitating calorie absorption and subsequent weight gain (42, 43). Additionally, the HFD group exhibited an elevated abundance of *Erysipelotrichaceae*, associated with high-fat diet consumption (44); *Coriobacteriaceae*, which tends to increase in tandem with the development of type 2 diabetes (45); and an active pathogen, *Fusobacteriaceae* (46). Conversely, treatments with IDCC 2102 and IDCC 4301 led to an enrichment in the proportion of *Enterococcus* and *Bifidobacterium*, respectively, indicating effective colonization. Furthermore, both treatments augmented the abundance of *S24-7*, *Lactobacillaceae*, and *Ruminococcaceae*, which are commensal bacteria prevalent in healthy canine microbiota (47, 48). These findings underscore the potential of IDCC 2102 and IDCC 4301 in positively shaping the gut microbiota composition.

Given the significant role of the gut microbiota in influencing host physiology through the production of bacterial metabolites, we conducted an analysis of bacterial metabolites and functional pathways to elucidate the mechanisms underlying the impact of IDCC 2102 and IDCC 4301 on the hyperlipidemic *C. elegans* and canine models. The *C. elegans* control group, under hyperlipidemic conditions, exhibited heightened levels of glycine-related components, indicating a connection to lipid accumulation. Remarkably, treatments with *E. faecium* IDCC 2102 and *B. lactis* IDCC 4301 were found to influence glycine synthesis, potentially contributing to an extended lifespan in *C. elegans*. The hyperlipidemic *C. elegans* strain displayed notable alterations in metabolites linked to fatty acid metabolism, alongside an accumulation of glycine. Importantly, the administration of probiotics reversed this metabolic profile. This reversal was marked by elevated levels of beneficial bacterial short-chain acids like 5-aminovaleric acid and acetic acid, along with an increase in the serotonin precursor L-tryptophan. Simultaneously, there was a decrease in the levels of glycine and its precursor molecules. These findings strongly suggest that probiotic treatment effectively regulates lipid accumulation and mitigates neurotoxicity in the hyperlipidemic *C. elegans* model.

In our canine models, we observed that IDCC 2102 and IDCC 4301 actively participated in energy-producing mechanisms, specifically involving canine pyruvate and glucose metabolism. Under conditions of obesity, marked by an excessive intake of energy from a high-fat diet, the incapacity to burn this surplus energy leads to its storage as triglycerides. Notably, treatments with IDCC 2102 and IDCC 4301 prompted the activation of the pyruvate mechanism, thereby promoting homeostasis in glucose metabolism and facilitating ATP production. Moreover, both IDCC 2102 and IDCC 4301 prompted the synthesis of substantial quantities of short-chain fatty acids, such as acetic acid and butyric acid, while decreasing choleric acid and adipic acid levels (49–51). Notably, IDCC 4301 exhibited a specialization in utilizing glucose metabolism, generating carboxylic acids like valeric acid and glutaric acid (52). Furthermore, our investigation revealed that *Enterococcus faecium* IDCC 2102 produced a noteworthy quantity of dopamine. Dopamine, a key catecholamine molecule, serves as a neurotransmitter regulating various animal behaviors, as established by prior research. In individuals with obesity, dopamine signaling indirectly influences pathological eating, leading to weight gain and glucose intolerance (53–55). Additionally, pyruvate has been identified as a protective factor for dopaminergic neurons (56).

**Fig 9:**
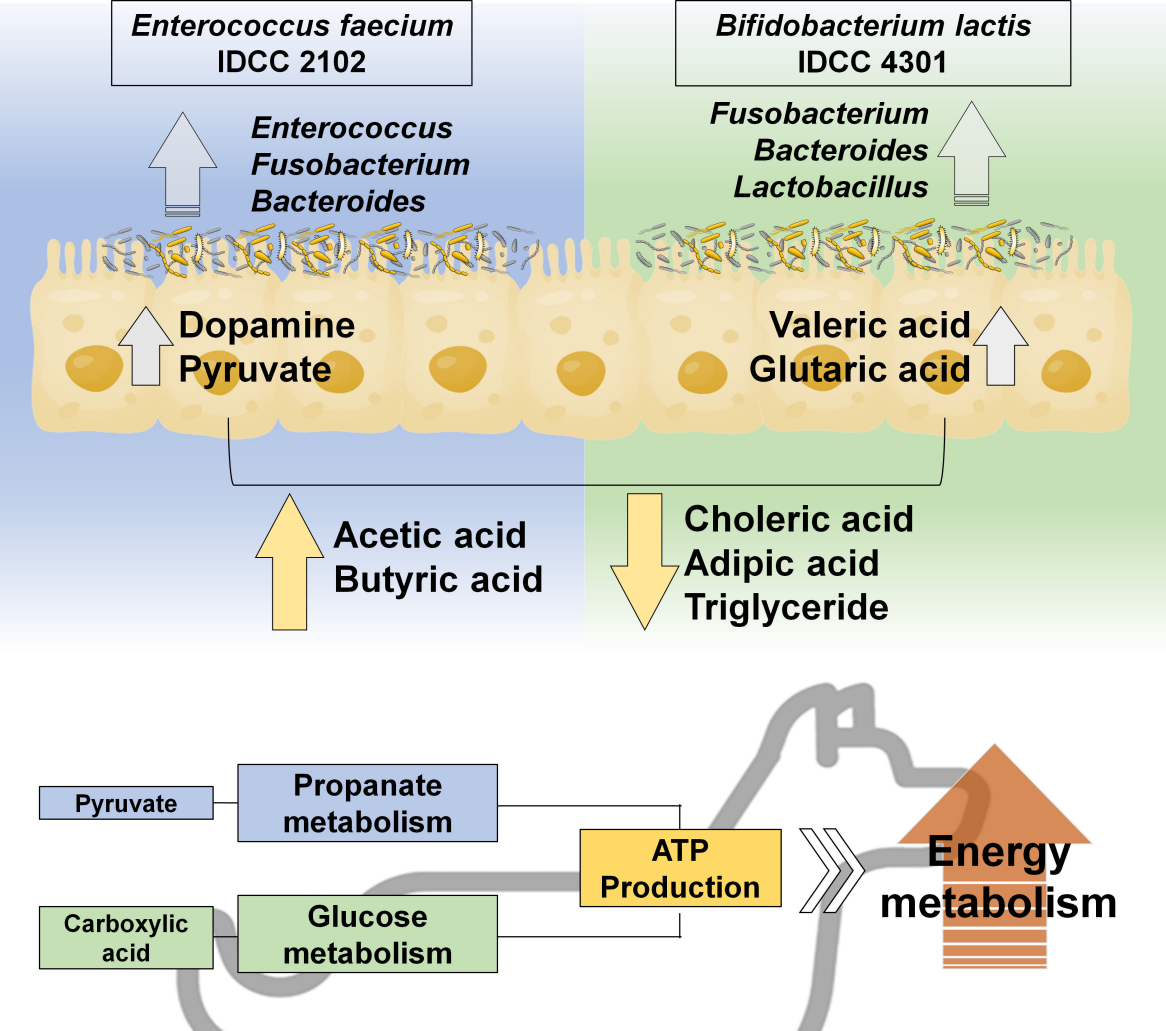
Graphical flow of probiotic-mediated alterations in obese canine metabolism. *Enterococcus faecium* IDCC 2102 and *Bifidobacterium lactis* IDCC 4301 affected commensal bacterial proliferation, and administering probiotics increased glycolysis and activated pyruvate metabolism in the body, which is related to propanate metabolism as a result of pyruvate metabolism activation boosting bacterial fatty acid production via dopamine and carboxylic acid specialized pathways, hence contributing to increased ATP synthesis and energy metabolism activity.

In summary, our investigation provides valuable insights into the intricate mechanisms through which *E. faecium* IDCC 2102 and *B. lactis* IDCC 4301 exert their influence on the host, with a particular emphasis on the context of obesity. These noble probiotics demonstrate the ability to activate energy-producing pathways, regulate the synthesis of short-chain fatty acids, and impact neurotransmitter production. This multifaceted approach holds promising potential for attenuating the effects of obesity and enhancing metabolic health in both canines and the *C. elegans* model. This sophisticated comprehension highlights the therapeutic capabilities of noble probiotics in addressing the intricate interplay of metabolic and neurobiological factors (Fig. 9).

Furthermore, this unveils potential applications for interventions aimed at enhancing metabolic health in various mammals, extending to humans, as evidenced by the *C. elegans* study, which serves as a human homolog model. However, the intricate nature of these interactions in metabolic diseases emphasizes the necessity for additional research to fully comprehend the broader implications and potential therapeutic applications of these findings in conditions related to obesity.

## MATERIALS AND METHODS

### Probiotic preparation

Ildong Bioscience Co., Ltd. (Gyeonggi-do, Republic of Korea) provided the probiotics for this experiment, *Enterococcus faecium* IDCC 2102 (IDCC 2102) and *Bifidobacterium lactis* IDCC 4301 (IDCC 4301), and they were cultured in de Man, Rogosa, and Sharpe (MRS; BD Difco, New Jersey, USA) at 37°C for 48 h under aerobic conditions. *Lacticaseibacillus rhamnosus* GG (LGG) was cultured in MRS at 37°C for 24 h under aerobic conditions. The bacterial cultures were freeze-dried and kept at −80°C before use.

### *C. elegans* culture conditions

*C. elegans fer15(b26)II;fem-1(hc17)IV* and *C. elegans* strain VS29 (vha-6p::GFP::dgat-2) were purchased from the Caenorhabditis Genetic Center (Minnesota, USA) and maintained on nematode growth medium (NGM) plates at 15°C. *E. coli* strain OP50 (OP50), the standard feed for *C. elegans*, was cultured in the Luria–Bertani medium (LB Broth, Miller; BD Difco) at 37°C for 24 h with 225 rpm shaking. The live bacterial lawn was prepared for *C. elegans* feeding by centrifuging the bacterial pellet at 13,000 rpm for 1 minute, washing it twice with sterile M9 buffer (3 g KH_2_PO_4_, 6 g Na_2_HPO_4_, and 5 g NaCl mixed in 1 L distilled water, autoclaved), and then adding 1 mL of 1 M MgSO_4_ (Sigma‒Aldrich, St. Louis, MO, USA). The bacterial pellet was then concentrated to a final concentration of 2.5 mg/L (wet weight) in M9 buffer and suspended on NGM plates [3.5 g Bacto Peptone (BD Difco), 3 g NaCl (Sigma‒Aldrich), and 20 g agar (BD Difco)] (11).

### Lifespan analysis

For the lifespan analysis, egg-bearing worms were bleached using a sodium hypochlorite‒sodium hydroxide solution (Sigma‒Aldrich), and the newborn worms were synchronized to L1 stage worms on NGM plates at 25°C (57). After 3 days, young adult L4 stage worms were plated on 35-mm-diameter NGM plates seeded with OP50, IDCC 2102, IDCC 4301, and LGG. The experiment was carried out in triplicate, and all worms were transplanted to new bacterial lawns on a daily basis until all worms died.

### Lipid staining for fat accumulation

To quantify the lipid accumulation of *C. elegans*, featured staining methods were applied to oil red O and Nile red O (58, 59). First, *C. elegans* strain VS29 was synchronized to the young adult L4 stage as described above and exposed to *E. coli* OP50, IDCC 2102, and IDCC 4301 for 48 h. The staining method followed bioprotocols using oil red O and Nile red solutions (Sigma‒Aldrich). The result was observed using an Olympus IX53 (Olympus Life Science, Tokyo, Japan), and the intensity was analyzed using i-Solution (IMT i-Solution Inc, New York, USA).

### Animal care and treatment

Beagles aged 2–4 years and weighing 9.0 ± 2.14 kg were purchased from Orient Bio (Gyeonggi-do, Republic of Korea) for the experiment (8 males and 12 females). They were given 2 weeks to acclimate to the breeding room atmosphere before the experiment began *ad libitum*. After acclimation, all subjects were randomly separated into four groups of five individuals based on body weight (9.24 ± 2.17 kg; *n* = 3 per group). The control group (cont) received a standard diet consisting of medium adult dry dog food (Royal Canin, Gard, France; ME (metabolizable energy) = 357.0 kcal/100 g), while the other groups were induced to develop obesity with a 22% high-fat diet (HFD; Jeilfeed, Seoul, Republic of Korea; ME (metabolizable energy) = 483.0 kcal/100 g). The probiotic groups were administered with 10^10^ CFU/day of IDCC 2102 and 10^10^ CFU/day of IDCC 4301 for 9 weeks, mixed with the high-fat diet. The nutritional compositions of normal chow and HFD are stated in Table S1. Each experimental animal was bred in a separate cage with an identity card during the experiment, including the acclimation period. The breeding room was maintained at a constant temperature (21°C ± 2°C), humidity (50% ± 20%), and photoperiod of 12 h (08:00–20:00). This animal experiment was conducted with the approval of the Institutional Animal Care and Use Committee at Chungnam National University (202109A-CNU-149).

### Physiological analysis

The body weight was obtained once a week following the commencement of the test at a specific time to observe the change in body weight during the experiment. To assess feed intake, the provided quantity of feed was measured daily at a specific time, and the residual amount was gauged 24 h later consistently throughout the experiment.

To monitor changes in the body shape of the beagle animal model as a result of a high-fat diet and probiotic feeding, the width of the abdomen and hips was measured once a week to score the body condition score, which is an index for determining the body shape of companion animals, and the score during the test period. Table S2 provides the body condition score index. The higher the degree of obesity, the higher the score, and the requirements for each score component are stated in Table S2 (60). The average value between the groups was calculated.

At the end of the 9-week experiment, all animals were fasted for 14 h, blood was collected and centrifuged (4°C, 3,000 rpm, 20 minutes), and serum was separated to analyze various physiological changes. The separated serum was kept at −70°C, and the experiment was carried out using ELISA. TNF-α, IL-1β, IL-6 (R&D Systems, Minnesota, USA), and C-reactive protein (Cusabio Technology LLC, Texas, USA) concentrations in the blood were analyzed to assess changes in inflammation-related cytokines. For the examination of energy metabolism regulators, the concentrations of leptin (Millipore, USA), adiponectin (ABclonal Technology, Massachusetts, USA), and malondialdehyde (Biocompare, California, USA) in the blood were measured according to the manufacturer’s instructions. Serum ALT, AST, HDL-C, TG, and TCHO were analyzed using a DRI-CHEM Analyzer (Fujifilm, Tokyo, Japan).

### Fecal metagenome analysis

After 9 weeks of experimentation, fecal samples were obtained from each subject, frozen at −70°C, and then used for the experiment to examine the intestinal microbial community’s response to feed. The first step was to extract the gDNA from each sample in accordance with the DNeasy PowerSoil Pro Kit’s manufacturer’s instructions (Qiagen, Hilden, Germany). Next, the V4 segment of the 16S ribosomal RNA gene was amplified using the extracted DNA sample, and Illumina iSeq 100 (Illumina, Inc., California, USA) was used for next-generation sequencing (61). The following is the primer sequence for the synthesis of the V4 region: 515F, TCGTCGGCAGCGTCAGATGTGTATAAGAGACAGGTGCCAGCMGCCGCGGTAA; 806R, GTCTCGTGGGCTCGGAGATGTGTATAAGAGACAGGGACTACHVGGGTWTCTAAT.

### Metabolite analysis

The pooled fecal samples of canines and *C. elegans* VS29 strains exposed to probiotics were weighed and diluted in methanol to a final concentration of 20 mg/mL on ice. To ensure significance, the metabolite analysis was repeated three times. The supernatant was filtered using a 0.2-µm pore size polyvinylidene fluoride syringe filter after centrifugation at 15,000 × *g* for 5 minutes at 4°C. Two hundred-microliter aliquots of the filtered supernatant were concentrated in a vacuum concentrator and kept at −81°C prior to derivatization and gas chromatography mass spectrometry (GC-MS) analysis. The extract was derivatized at 30°C for 90 minutes with 30 µL of 20 mg/mL methoxyamine hydrochloride in pyridine (Sigma‒Aldrich), followed by 50 µL of N,O-bis(trimethylsilyl)trifluoroacetamide (Sigma‒Aldrich). As an internal standard, fluoranthene was added to the extract. A Thermo Trace 1310 GC (Waltham, MA, USA) linked to a Thermo ISQ LT single quadrupole mass spectrometer was used for the GC-MS analysis (Waltham, MA, USA). A 60-µm long DB-5MS column with an inner diameter of 0.2 mm was used. The derivatives were separated using a 0.25 m film thickness (Agilent, Santa Clara, CA, USA). The sample was injected at 300°C with a split ratio of 1:60, and the helium split flow was 90 mL/min for analysis. The metabolites were separated using 1.5 mL of continuous flow helium and an oven ramp of 50°C (2 minute hold) to 180°C (8 minute hold) at 5°C/min, 210°C at 2.5°C/min, and 325°C (10 minute hold) at 5°C/min. The mass spectra peaked at a rate of 5 spectra/s throughout the scan in the range of 35–650 m/z. The ionization mode was exposed to electron impact with the ion source temperature adjusted to 270°C. Thermo Xcalibur software was used to analyze the spectral peaks, and the metabolites were identified by matching the mass spectra and retention indices of the NIST Mass spectral search tool (version 2.0, Gaithersburg, MD, USA) (62, 63).

### Correlation analysis for the metagenome and metabolome

The Shannon and Chao indices of bacterial alpha diversity were analyzed using a non-parametric one-way analysis of variance (Kruskal–Wallis test) and Tukey’s *post hoc* analysis if a significant difference (*P* < 0.05) was found. Welch’s *t*-test was used to compare the differences in the relative abundance of bacterial composition (64, 65). The correlation between the microbiota and metabolites was conducted using the M^2^IA (66). Microbiome–metadata correlation analyses and analyses of the different levels of microbial communities by genus were performed. A network was used to examine the relationship between the functional metabolites and the bacteria. The pink nodes are the functional metabolites, and the other nodes of bacteria were grouped by color (functional false discovery rate <0.05). To discover functional pathways in the fecal microbiome, PICRUSt and KEGG (level 2) were utilized to predict the existence of functional genes in the sample. PICRUSt was completed via an online application (67, 68). Using STAMP v0.2.1.3 and an extended error bar plot, the difference in the relative abundance of bacteria and significantly different KEGG pathways within groups was calculated.

A volcano plot and principal component analysis (PCA) of metabolomics data were processed using GraphPad Prism 7.0.4 (GraphPad Software, California, USA), and heatmap analysis was processed using MetaboAnalyst 5.0 (69).

### Statistics

All data points used for this study were analyzed in triplicate, and the data are expressed as the mean ± standard deviation. Significant differences were determined according to Student’s *t*-test and one-way ANOVA using GraphPad Prism 7.0.4 (GraphPad Software) and SigmaPlot 13 (Systat Software, California, USA) followed by Tukey’s *post hoc* test. *C. elegans* survival analysis was performed using the Kaplan–Meier method, and the significance of differences between survival curves was determined using the log-rank test (STATA6; STATA, College Station, TX, USA).

## Data Availability

The fecal metagenome dataset, accessible via NCBI BioProject under PRJNA898689.

## SUPPLEMENTAL MATERIAL

Fig. S1 and S2 and Tables S1 and S2 spectrum.02552-23-s0001.docx Table S1 (Nutritional compositions of normal chow and high fat diet), Table S2 (The scoring index of body condition scores for medium-sized canines), Figure S1 (Probiotics treatment regulated glycine metabolism in hyperlipidemic *C. elegans* VS29), and Figure S2 (Measuring the midsection circumferences for the canine body condition scores).

ASM does not own the copyrights to Supplemental Material that may be linked to, or accessed through, an article. The authors have granted ASM a non-exclusive, world-wide license to publish the Supplemental Material files. Please contact the corresponding author directly for reuse.
