# Supplementary material for: Dietary supplementation with probiotics promotes weight loss by reshaping the gut microbiome and energy metabolism in obese dogs
Source: Microbiol Spectr. 2024 Jan 25;12(3):e02552-23. doi: 10.1128/spectrum.02552-23 (PMC10913549; doi:10.1128/spectrum.02552-23)

SUPPORTING INFORMATION

**Dietary supplementation with probiotics promotes weight loss by reshaping the gut microbiome and energy metabolism in obese dogs**

An Na Kang^a†^, Min-Jin Kwak^a^_,_ Daniel Lee^a^, Jeong Jae Lee^b^, Min Kyu Kim^c^, Minho Song^c^, Minjee Lee^d^, Jungwoo Yang^d^, Sangnam Oh^e*^, and Younghoon Kim^a*^

^a^Department of Agricultural Biotechnology and Research Institute of Agriculture and Life Science, Seoul National University, Seoul 08826, Korea

^b^Institute of Agricultural Science and Technology, Kyungpook National University, Daegu 41566, Korea

^c^Division of Animal and Dairy Science, Chungnam National University, Daejeon 34134, Korea

^d^Ildong Bioscience, Pyeongtaek-si, Gyeonggi-do, 17957, Republic of Korea

^e^Department of Functional Food and Biotechnology, Jeonju University, Jeonju 55069, Korea

*To whom correspondence should be addressed: osangnam@jj.ac.kr and ykeys2584@snu.ac.kr

**Supplementary Table S1.** Nutritional compositions of normal chow and high fat diet.

|  | **Normal chow**  **(medium adult dry dog food; Royal canin)** | **High-fat diet**  **(Jeilfeed)** |
| --- | --- | --- |
|  | **% / 100g** | **% / 100g** |
| Water | 9.5 | 6.1 |
| Fat | 16.0 | 33.1 |
| Protein | 23.0 | 29.3 |
| Starch | 38.4 | 13.4 |
| Total fiber | 6.7 | 10.1 |
| Ash | 6.4 | 8.0 |
| ME (kcal/100g) | 357.0 | 483.0 |
|  | **% Energy** | **% Energy** |
| Fat | 34 | 63 |
| Protein | 30 | 25 |
| Starch | 36 | 12 |

**Supplementary Table S2.** The scoring index of body condition scores for medium-sized canines.

| **Obesity factor** | **Score** | **Body shape** |
| --- | --- | --- |
| Underweight | 1 | The ribs, spine, and lumbar bones are visible to the naked eye and the stomach's curvature is plainly evident from the side. |
|  | 2 | It is difficult to notice the bones of the upper back with the naked eye, yet the curve of the lower back and the stomach are plainly evident when viewed from the side. |
|  | 3 | When viewed from the side, the abdomen rises in a smooth curve and fat is noticeable. |
| Ideal | 4 | When the lower back and lateral abdominal curvature are negligible, and the fat and ribs are perceptible. |
|  | 5 | There is no curvature of the lower back and just a modest curve of the abdominal sides. |
| Above ideal | 6 | Abdominal fat has collected to the point where the ribs are no longer visible and the lateral abdomen has a little inward curve. |
| Overweight | 7 | The ribs are not easily perceptible, and the lateral abdomen has a reversed curvature. |
| Obese | 8 | The accumulation of fat around the waist is visible from above, and the accumulation of lateral abdominal fat is excessive, causing the ribs to be touched with force. |
|  | 9 | When viewed from above, the accumulation of fat around the waist is evident, the lateral abdominal flexion is reversed, the abdominal fat accumulation is severe, so the skin is pulled tightly, and there are fat-related wrinkles around the neck. |

**Supplementary Figure S1. Probiotics treatment regulated glycine metabolism in hyperlipidemic C. elegans VS29**


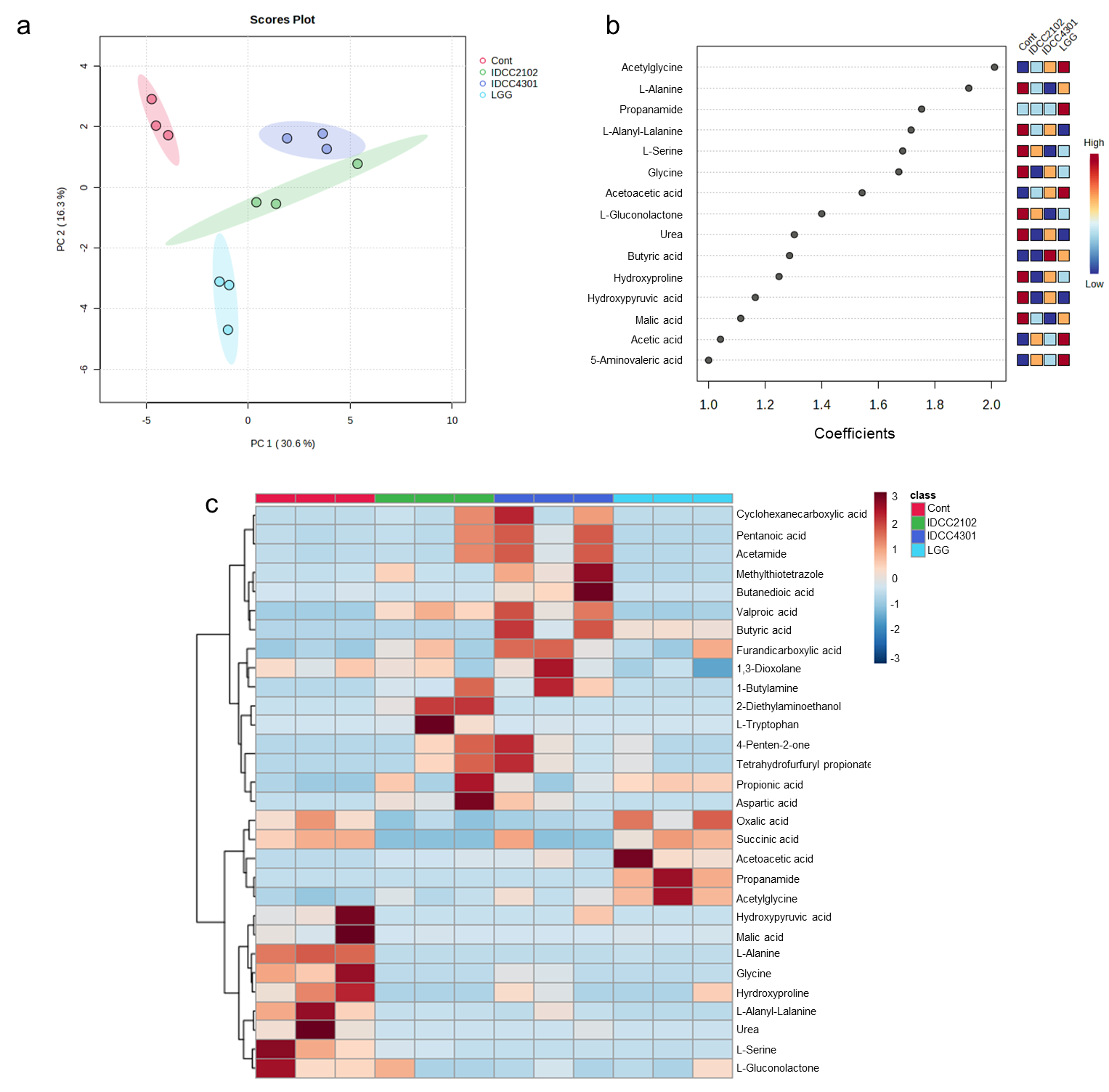


**Supplementary Figure S2.** Measuring the midsection circumferences for the canine body condition scores.


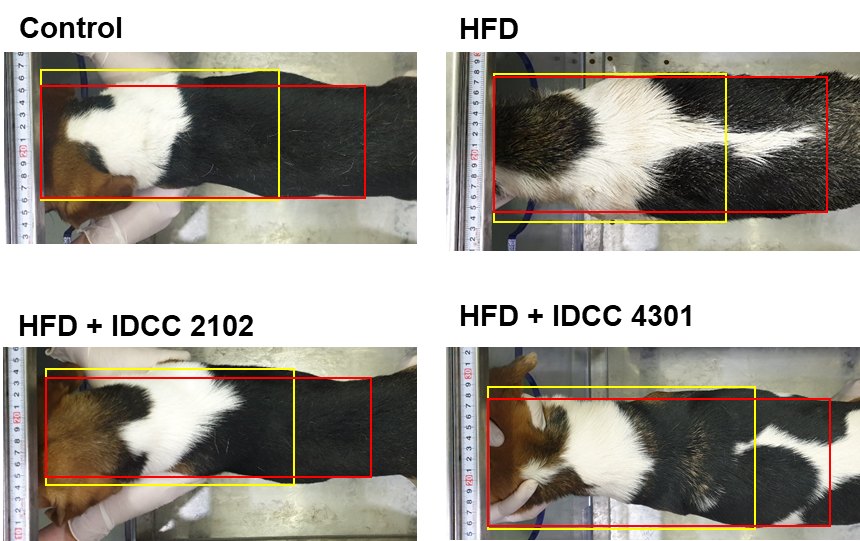

Supplement: Fig. S1 and S2 and Tables S1 and S2 — Table S1 (Nutritional compositions of normal chow and high fat diet), Table S2 (The scoring index of body condition scores for medium-sized canines), Figure S1 (Probiotics treatment regulated glycine metabolism in hyperlipidemic C. elegans VS29), and Figure S2 (Measuring the midsection circumferences for the canine body condition scores). [file spectrum.02552-23-s0001.docx]
